# Machine Learning-Based Prognosis Prediction in Glioblastoma Multiforme Patients by Integrating Clinical Data with Multimodal Radiomics †

**DOI:** 10.3390/diagnostics16040512

**Published:** 2026-02-08

**Authors:** Mohan Huang, Man Kiu Chan, Ka Lung Cheng, Pak Yuen Hui, Shing Yau Tam

**Affiliations:** School of Medical and Health Sciences, Tung Wah College, Kowloon, Hong Kong SAR, China; mhhuang@twc.edu.hk (M.H.);

**Keywords:** glioblastoma multiforme, hypoxia, machine learning, multimodal imaging, prognostic model, radiomics

## Abstract

**Objectives**: Glioblastoma multiforme (GBM) is considered the most aggressive primary brain tumor, which often exhibits tumor heterogeneity. Hypoxia is a key aspect of intratumoral heterogeneity that contributes to poor prognosis in GBM. In this study, we aimed to develop machine learning (ML) models using radiomics and clinical features for the prediction of one-year survival for GBM. **Methods**: Data from 35 patients in the ACRIN 6684 trial, including fluoromisonidazole (FMISO)-positron emission tomography (PET), magnetic resonance (MR) (T1, T2, and fluid-attenuated inversion recovery (FLAIR)) images, and clinical information, were retrieved from The Cancer Imaging Archive (TCIA). Three ML algorithms, namely, support vector machine (SVM), random forest (RF), and linear regression (LR), were utilized to analyze selected features. Receiver-operating characteristic (ROC) curves were utilized to evaluate the predictive performance of the models. Several statistical analyses, namely, the permutation test, the permutation importance of selected features, Fisher’s exact test, and the unpaired *t*-test, were performed to analyze the models and features. **Results**: FMISO achieved the best performance in radiomics models, with an area under the curve (AUC) of 0.870. The clinical data model achieved the best performance of all models, with an AUC of 0.921, outperforming the combined all sequential forward selection (SFS) model (AUC: 0.862). Female sex (*p* = 0.030) and younger age (*p* = 0.0043) were significantly associated with better prognosis. **Conclusions**: Our proposed models have the potential to predict the one-year survival of GBM and facilitate personalized therapy. Future studies with a larger sample size are needed to confirm the generalizability of the models.

## 1. Introduction

Glioblastoma multiforme (GBM), classified as a grade IV glioma, is the most common primary brain tumor in adults, accounting for approximately 15% of all central nervous system malignancies [1]. The incidence rate of GBM ranges from 0.59 to 5 cases per 100,000 individuals and has been rising in many countries [2]. Recurrence is exceedingly common in GBM patients, occurring in 75–90% of cases [3]. The standard management for GBM is debulking surgery [4], followed by concurrent daily radiation therapy (RT) and oral chemotherapy using temozolomide (TMZ) as adjuvant therapies for 6 weeks, and then maintenance TMZ is prescribed 5 days a month for 6 months [5]. However, GBM patients experience a median survival of only around 14 to 15 months from diagnosis worldwide [6].

GBM has been considered the most aggressive primary brain tumor. This is because GBM tends to infiltrate extensively into normal brain tissue and exhibits an immunosuppressive microenvironment [7]. Also, it often displays both intertumor and intratumor heterogeneity. Intertumor heterogeneity refers to the differences among patients. GBM is classified according to molecular subtypes, which influence prognosis and treatment response. Intratumor heterogeneity, meanwhile, is defined as the difference within a tumor. The core region in GBM is often necrotic and hypoxic, which is linked to poor prognosis [8].

Hypoxia is a widespread feature of solid tumors and is observed in 90% of solid tumors [9]. It occurs due to the rapid tumor growth that exceeds the oxygen supply, combined with compromised blood flow resulting from the formation of abnormal blood vessels supplying the tumor [10]. It is known that hypoxia promotes tumor growth by the activation of hypoxia-inducible factors (HIFs). HIFs regulate the proliferation of cancer cells by controlling genes for apoptosis and stress response, as well as the angiogenesis signaling pathway [9]. Moreover, tumor hypoxia contributes to resistance to RT and chemotherapy [11]. Regarding radio-resistance, oxygen can stabilize the damage of DNA by reacting with fractured DNA strands to form peroxide. Tumor cells are likely to be repaired from sublethal damage in hypoxic environments [12]. Regarding chemo-resistance, hypoxia upregulates the multidrug resistance 1 (MDR1) gene to reduce the intracellular concentration of chemotherapy drugs [13]. As tumor hypoxia has evident links to poor prognosis, several methods have emerged to detect and measure hypoxia. A polarographic oxygen electrode is widely perceived as the gold standard since it can provide the most accurate oxygen tension. However, due to its invasiveness, a polarographic oxygen electrode is rarely used in clinical practice [14], whereas a positron emission tomography (PET) scan using a radioactive tracer called fluoromisonidazole (FMISO) is a non-invasive measurement that can provide a standardized uptake value (SUV) to assess hypoxia severity [15]. Although these techniques provide quantitative assessments of tumor hypoxia, they cannot reliably establish a definitive prognosis for individual patients’ overall survival.

Radiomics is an innovative field in imaging analysis that involves the extraction of radiomics features from regions of interest (ROIs) in medical images. Radiomics features can be divided into five main groups, namely, histogram-based features, texture-based features, mode-based features, transform-based features, and shape-based features [16]. In recent years, machine learning (ML) algorithms have been combined with radiomics to build models for making predictions on cancer diagnosis, treatment response, and prognosis [17]. Previous studies employed radiomics-based approaches, integrating imaging and clinical information, to develop ML models for predicting overall survival in GBM [18,19,20]. Muzi et al. [18] found that hypoxic volume and SUV peak are the two independent predictors of prognostic outcome in GBM patients by studying the FMISO images.

Despite these advances, important challenges remain in translating radiomics-based models into clinically meaningful prognostic tools for GBM. It is noted that no published studies have investigated the integration of FMISO and magnetic resonance (MR) radiomics with clinical data in ML for GBM patients’ overall survival. Most existing studies rely on single-modality imaging or radiomics alone, while the integration of hypoxia-sensitive PET, multiparametric MRI, and clinical variables within a unified machine learning framework remains limited. Addressing this gap is essential for improving risk stratification and clinical relevance.

To fill this research gap, we aim to develop a radiomics ML model to predict the one-year survival of GBM patients. The main contributions of this study are summarized as follows:

- We developed and validated machine learning models for one-year survival prediction in GBM using FMISO-PET, multiparametric MRI radiomics, and clinical data.
- We systematically compared the prognostic performance of individual imaging modalities, clinical variables, and their combined models within a unified LOOCV framework.
- We evaluated the relative importance of hypoxia-related radiomics features and clinical factors to enhance the interpretability and clinical relevance of the proposed models.
- This study provides evidence supporting the potential role of hypoxia-informed radiomics in personalized prognostic assessment for GBM.

## 2. Materials and Methods

### 2.1. Data Acquisition

Datasets were acquired in the American College of Radiology Imaging Network (ACRIN) 6684 clinical trial which is a prospective multicenter imaging study, from The Cancer Imaging Archive (TCIA) [21]. The inclusion criteria of the ACRIN 6684 trial are as follows: All participants must be adults who have provided written informed consent. The disease management plan for all participants must include surgery followed by concurrent conventional fractionated radiotherapy (RT) with oral temozolomide. Participants must have a residual tumor after surgery, and their Karnofsky Performance Status (KPS) must exceed 60. The exclusion criteria of the ACRIN 6684 trial are as follows: Patients are pregnant or breastfeeding. Patients are unfit for a magnetic resonance imaging (MRI) exam due to metallic implants, claustrophobia and allergies to contrast agents. Patients are willing to provide clinical data on additional treatment modalities such as immunotherapy. Patients suffer from sickle cell disease, renal failure or other serious systemic complications [22]. There are 45 patients who participated in the ACRIN 6684 trial. The datasets contain medical images, including FMISO-PET, MRI, and computed tomography, as well as clinical data, including age, gender, race, KPS, tumor hypoxic volume, SUV (maximum, minimum, and average), and other relevant variables.

### 2.2. Selection of the Study Cohort

The datasets were further screened to align with the objectives of our study. The screening criteria were as follows: The clinical data of all selected patients must contain the number of days from the base date to the date confirmed alive. All selected patients must be present with FMISO-PET, T1-weighted MRI, T2-weighted MRI, and fluid-attenuated inversion recovery (FLAIR) MRI.

### 2.3. Imaging Preprocessing and Segmentation

Image preprocessing is the first step of radiomics to standardize medical images. This can ensure that extracted radiomics features are comparable and reproducible [23]. In this study, images were resampled to an isotropic voxel spacing of 1.0 × 1.0 × 1.0 mm^3^ by using B-spline interpolation for MR images and trilinear interpolation for PET images. Also, discretization of images was carried out to set a fixed bin width of 0.2 for all images. All these procedures were completed in 3D slicer, version 3.8.0. These methods preserve image texture properties critical for radiomic analysis.

Segmentation is a process in which ROIs are delineated so that regions linked to prognosis are focused on for feature extraction. In this study, regarding FMISO-PET, regions with increased uptake, which indicate hypoxic regions, were considered as ROIs. Regarding MRI, tumor boundaries were defined as ROIs. Several extensions in 3D slicer, version 3.8.0, were utilized to facilitate the process of segmentation, namely, ‘Slicer Radiomics’, ‘PET Tumor Segmentation’, ‘PET DICOM Extension’, and ‘PET-IndiC’.

### 2.4. Feature Extraction and Normalization

With ROIs contoured, feature extraction was carried out to capture radiomics features for model building. This procedure was performed using another extension in 3D slicer (v3.8.0), which was PyRadiomics. A total of 107 radiomics features were extracted for each image. These radiomics features could be divided into 7 groups, namely, shape, first order, gray level cooccurrence matrix (GLCM), gray level dependence matrix (GLDM), gray level run length matrix (GLRLM), gray level size zone matrix (GLSZM), and neighborhood gray tone difference matrix (NGTDM).

Feature normalization refers to the process of scaling radiomic features. This procedure prevents features with large numerical ranges from dominating model training, thereby improving both training efficiency and model performance [24]. In this study, Z-score standardization was performed using Python (v3.11.4), whereby all continuous radiomic features were normalized using Z-score standardization: z = (x − μ)/σ, where μ and σ are the mean and standard deviation computed from the training set in each LOOCV fold. This ensures features have zero mean and unit variance, improving model convergence and coefficient comparability.

### 2.5. Feature Selection

Sequential forward selection (SFS) was implemented using the SequentialFeatureSelector class in scikit-learn (v1.3.0). For each modality-specific model, SFS started with an empty feature set and iteratively added the feature that maximized the cross-validated AUC (using internal 5-fold CV during SFS). The search terminated when adding any remaining feature no longer improved the AUC by at least 0.01, or when the number of selected features reached a pre-defined upper bound of min (10, *n*_samples/3) to prevent overfitting. This iterative approach identifies a parsimonious subset of *N* features that maximizes the model’s discriminative power while minimizing overfitting. The number of features selected through SFS enforced a minimum selection of 2 features, with upper limits defined by sample size constraints.

### 2.6. Model Development and Validation

Given the limited sample size (*n* = 35), this study employed 3 ML algorithms, including support vector machine (SVM), random forest (RF) and linear regression (LR) for model development. We opted for interpretable classical machine learning algorithms—namely, support vector machine (SVM), random forest (RF), and linear regression (LR)—rather than deep learning or survival-aware models (e.g., Cox-based neural networks) as these complex models typically require substantially larger datasets to avoid severe overfitting and ensure stable convergence. Our choice prioritized model stability, interpretability, and robustness under data scarcity, aligning with best practices for small-sample radiomics studies [17]. Leave-one-out cross-validation (LOOCV) [25] was carried out to obtain the ROC curves and three performance parameters, including accuracy, precision and recall, to evaluate the performance among models. In this study, 35 patients were included. Therefore, LOOCV created 35 iterations for each model. In each iteration, 34 samples were used for training, while the single held-out sample was used as validation. The result of all 35 iterations was averaged to create the averaged ROC curves. Additionally, bootstrapping was carried out to obtain 95% confidence intervals of the AUC for the estimation of variability.

### 2.7. Statistical Analysis

Several statistical analyses were performed to evaluate the models’ performance and interpret the results. First, a permutation test was conducted to assess whether the models’ performance was statistically significant. A permutation *p*-value of 0.01 was employed to ensure minimal overfitting and increase confidence in replicability. After passing the permutation test, permutation importance was used to identify the most influential features within the best-performing ML model of each modality. Furthermore, to confirm the association between the selected clinical feature and one-year survival, Fisher’s exact test and an unpaired *t*-test were employed for categorical and continuous data, respectively. Data are reported as means with a 95% confidence interval (CI) or standard deviation.

## 3. Results

**Figure 1:**
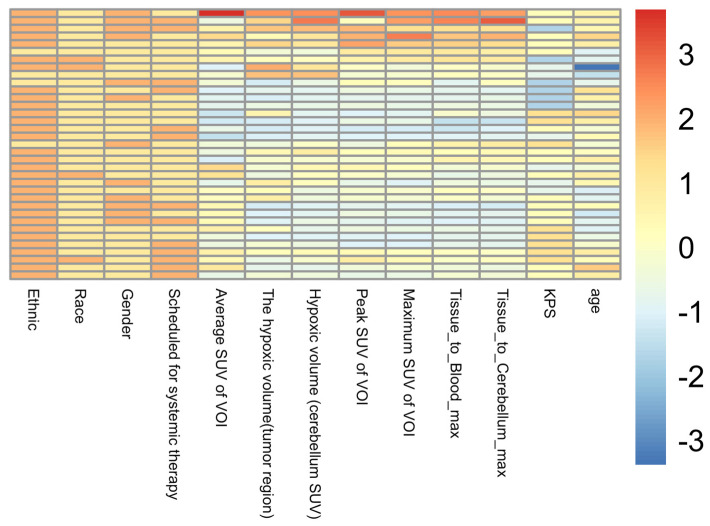
Heatmap of demographic characteristics.

A total of 35 patients with GBM were included in this study, comprising 12 females and 23 males. Of these, 16 patients died within one year of the baseline date of the ACRIN 6684 trial (indicated as dead), while 19 patients survived beyond one year (indicated as alive). The patient demographic information is summarized in Table 1, and a heatmap of the demographic characteristics is shown in Figure 1.

ROC curves were used to evaluate the prognostic performance of GBM across different models, including radiomic models of the individual imaging modalities (FMISO, T1, T2, and FLAIR), clinical data model, and the combined all SFS model using the four imaging modalities. Area under the curve (AUC) values derived from ROC curves were used to represent the overall performance of each model. Additionally, accuracy, precision and recall were employed to assess model performance at specific classification thresholds (Table 2). The highest-performing model in each imaging modality except FMISO-SVM, FMISO-LR, FLAIR-SVM and FLAIR-LR showed significant predictive power (*p* < 0.01), which means the performance of the models was statistically significant and not due to random chance.

### 3.1. FMISO

**Figure 2:**
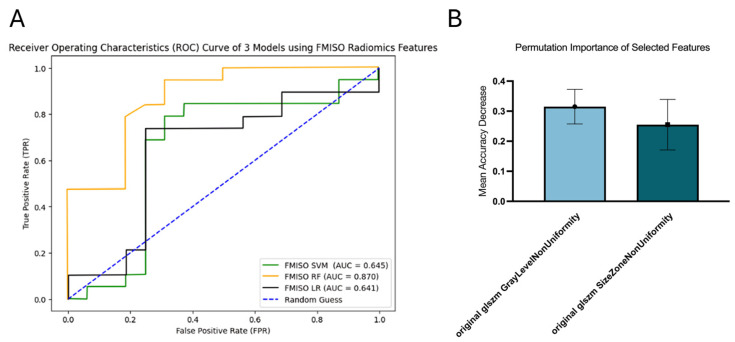
ROC curves of FMISO model (**A**) and permutation importance of selected features in FMISO RF (**B**).

RF demonstrated the best performance in survival prediction with an AUC of 0.870 (95% CI [0.734, 0.970]). SVM and LR showed similar results, with AUCs of 0.645 (95% CI [0.441, 0.853]) and 0.641 (95% CI [0.444, 0.840]), respectively (Figure 2A). Features selected for the RF model included gray-level non-uniformity (GLNU) and size zone non-uniformity (SZNU), with GLNU exhibiting the highest permutation importance of 0.3152 ± 0.0578 (Figure 2B).

### 3.2. T1

**Figure 3:**
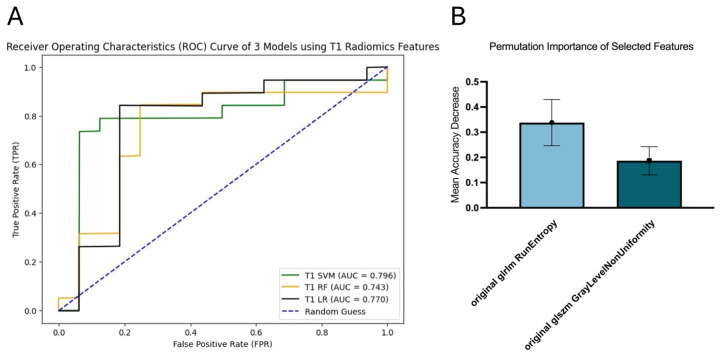
ROC curves of T1 model (**A**) and permutation importance of selected features in T1 SVM (**B**).

SVM achieved the best performance for survival prediction with an AUC of 0.796 (95% CI [0.608, 0.945]), while RF exhibited the lowest performance with an AUC of 0.743 (95% CI [0.553, 0.902]). Meanwhile, LR had an AUC of 0.770 (95% CI [0.576, 0.920]) (Figure 3A). Two radiomics features were selected for SVM, namely, run entropy (RE) and GLNU. RE obtained the highest permutation importance of 0.3381 ± 0.0916 (Figure 3B).

### 3.3. T2

**Figure 4:**
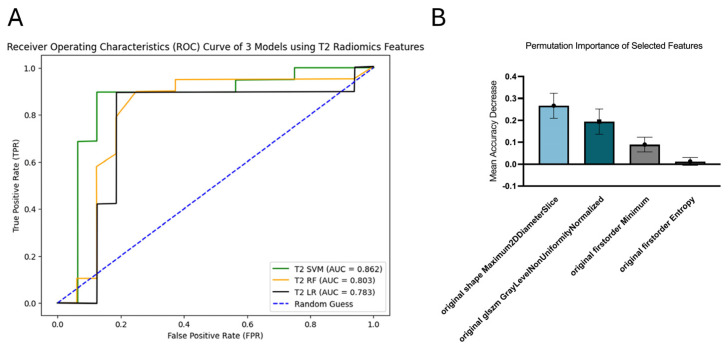
ROC curves of T2 model (**A**) and permutation importance of selected features in T2 SVM (**B**).

SVM achieved the highest performance for survival prediction, with an AUC of 0.862 (95% CI [0.709, 0.985]). In comparison, RF had an AUC of 0.803 (95% CI [0.610, 0.953]), while LR recorded the lowest AUC of 0.783 (95% CI [0.601, 0.935]) (Figure 4A). Four radiomics features were selected for the SVM model, including maximum 2D diameter slice (M2DS), gray-level non-uniformity normalized, first-order entropy and minimum. Among these, M2DS exhibited the highest permutation importance of 0.2667 ± 0.0568 (Figure 4B).

### 3.4. FLAIR

**Figure 5:**
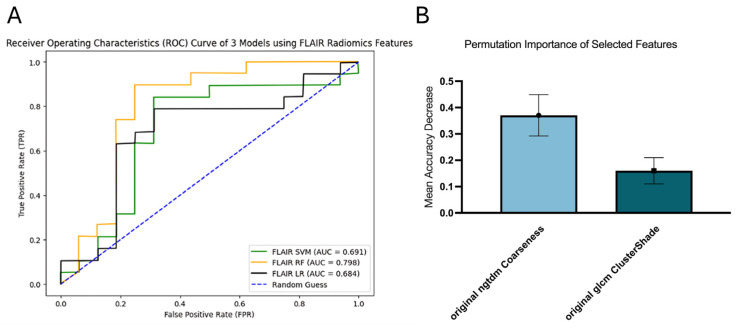
ROC curves of FLAIR model (**A**) and permutation importance of selected features in FLAIR RF (**B**).

RF demonstrated the best performance in survival prediction, with an AUC of 0.798 (95% CI [0.618, 0.959]). In contrast, SVM and LR achieved lower AUCs, both below 0.7, with AUCs of 0.691 (95% CI [0.477, 0.874]) and 0.684 (95% CI [0.484, 0.863]), respectively (Figure 5A). Two radiomic features were selected for the RF model, including original NGTDM Coarseness and original GLCM ClusterShade. Among these, original NGTDM Coarseness had the highest permutation importance of 0.3705 ± 0.0786 (Figure 5B).

### 3.5. Clinical Data-Based Model

**Figure 6:**
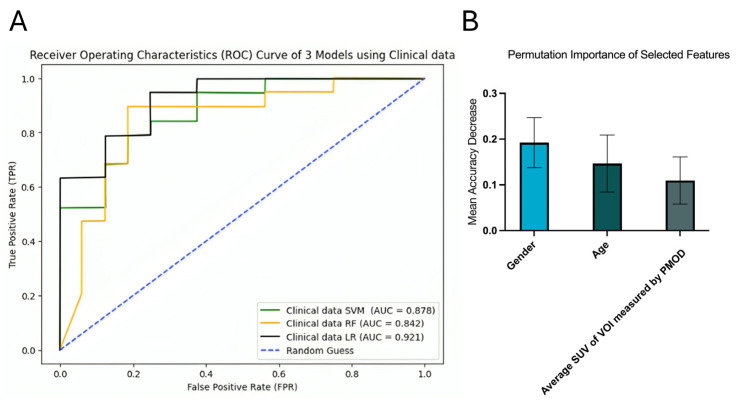
ROC curves of clinical data model (**A**) and permutation importance of selected features in clinical data LR (**B**).

LR demonstrated the best performance for survival prediction, with an AUC of 0.921 (95% CI [0.820, 0.986]). It outperformed SVM and RF, which had AUCs of 0.878 (95% CI [0.742, 0.971]) and 0.842 (95% CI [0.673, 0.976]), respectively (Figure 6A). Three clinical features were selected for the LR model, including age, gender, and average SUV. Among these, gender had the highest permutation importance of 0.1924 ± 0.0547 (Figure 6B).

There was a statistically significant association between gender and survival (*p*-value = 0.030) using Fisher’s exact test, indicating that males had a higher chance of dying within one year. For age, the unpaired *t*-test found a significant difference between the surviving and deceased groups after one year (*p*-value = 0.0043), showing that older patients had a higher chance of mortality.

### 3.6. Combined All Sequential Forward Selection (SFS) Model

**Figure 7:**
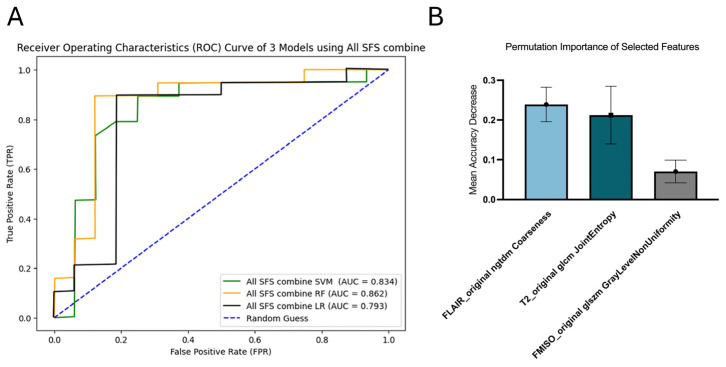
ROC curves of combined all SFS model (**A**) and permutation importance of selected features in combined all SFS RF (**B**).

RF demonstrated the highest predictive performance, with an AUC of 0.862 (95% CI [0.701, 0.989]). SVM achieved the second-best performance, with an AUC of 0.834 (95% CI [0.661, 0.966]). The AUC for LR was 0.793 (95% CI [0.595, 0.947]) (Figure 7A). Three features were selected for the RF model: coarseness from FLAIR images, joint entropy from T2 images, and GLNU from FMISO images. Among them, coarseness from FLAIR had the highest permutation importance of 0.2390 ± 0.0433 (Figure 7B).

## 4. Discussion

Our results demonstrated that the FMISO model achieved the best overall performance among the radiomics models for survival prediction, surpassing the T1, T2, and FLAIR models. In addition to having the highest AUC of 0.870, the FMISO model also obtained the highest recall, which was 0.947. Recall measures how frequently an ML model correctly identifies positive cases out of all actual positive cases. It is calculated as the number of true positives divided by the sum of true positives and false negatives [26]. In this study, false negatives refer to patients who were incorrectly classified as surviving beyond one year from the baseline date of the ACRIN 8864 trial. The high recall of the FMISO model indicates its ability to minimize false negatives, which is critical for survival prediction. This implies that the FMISO model could effectively identify most patients at high risk of poor survival, enabling early clinical decision making for both patients and physicians.

Our results indicated that the clinical data model achieved the best predictive performance among all models, with an AUC of 0.921. This suggests that the clinical data model outperformed the combined all SFS model (AUC = 0.862). Though direct comparison is challenging due to differing endpoints (time-to-event vs. binary survival) and evaluation metrics (C-index vs. AUC), notably, this finding differs from the study by Muzi et al. [18], which reported that a model integrating radiomics features with clinical data (C-index: 0.774) demonstrated better predictive performance than the clinical data model alone (C-index: 0.722). There are two potential reasons to explain this discrepancy. First, the difference may stem from the disparate ML algorithms employed. Muzi et al. [18] utilized the Cox proportional hazard model to focus on time-to-event outcomes [27], as their goal was to estimate survival time in GBM patients. In contrast, our study focused on predicting one-year survival, a binary outcome, using SVM, RF, and LR algorithms. Second, Muzi et al. [18] only incorporated FMISO radiomics features to build their hybrid model, whereas our study included FMISO, T1, T2, and FLAIR radiomics features for model selection. With the wide diversity of features provided, this could result in overfitting of the combined all SFS model, thus decreasing the performance of survival prediction. The underperformance of the combined SFS model relative to the clinical-only model may further support the hypothesis that feature redundancy and dimensionality overwhelm the limited statistical power of our cohort. Dimensionality reduction via PCA or autoencoders might alleviate this, but requires external validation.

The superior performance of the clinical-only model (AUC = 0.921) over all radiomic and hybrid models raises important questions about the incremental value of radiomics in this context. One plausible explanation is that age and gender—readily available, highly reproducible clinical variables—are strong baseline prognosticators in GBM, whereas radiomic features may introduce noise without a sufficient sample size to discern the true signal. In this study, Fisher’s exact test and an unpaired *t*-test were conducted to analyze the two highest permutation importance of selected features: gender and age. Fisher’s exact test showed that there was a significant association between gender and one-year survival of GBM, as female was correlated with better prognosis. The unpaired *t*-test demonstrated that there was a significant association between age and one-year survival of GBM, as younger age was correlated with better prognosis. It is noteworthy that our clinical data model could be suitable for application in clinical practice, as the collection of clinical data, such as age and gender, is convenient and routinely assessed. Therefore, the clinical data model is recommended for preliminary risk stratification, providing an initial insight into patient survival outcomes in clinical settings. Moreover, the observed strong association of female sex with better survival (*p* = 0.030) must be interpreted cautiously due to a significant gender imbalance (23 males vs. 12 females). This imbalance may inflate the apparent importance of gender, as confirmed by its high permutation importance (0.192). While biological differences in GBM outcomes by sex are documented [28], our finding may partly reflect sampling bias. Future studies should employ balanced cohorts or apply resampling techniques (e.g., SMOTE) to mitigate this confounder. Nevertheless, there are factors that may cause potential bias and affect the outcomes of the model. Firstly, some of the medical images in the ACRIN 8864 trial exhibited poor image quality, such as a low image resolution of some FMISO images and the presence of artifacts in some MR images. This might potentially hinder the contouring of ROIs. Some of the regions that are linked to prognosis could be missed, thus reducing the quality of extracted radiomics features [29]. Meanwhile, it is noted that there were 23 male patients and 12 female patients among 35 patients. The ratio of male patients to female patients was approximately 2:1. With the imbalanced dataset, this might contribute to the overestimation of the permutation importance of gender in the clinical data model. Under-sampling from the majority class could be utilized to reduce this bias.

A major limitation of our study is the small sample size (*n* = 35), which inherently restricts the generalizability of our findings, particularly when modeling high-dimensional radiomic feature spaces. To address this issue, we employed LOOCV. Unlike k-fold cross-validation, which divides the data into k folds and alternately trains and tests on (k − 1) folds, LOOCV trains and validates each individual sample [25]. This approach maximizes the use of available data and helps mitigate the challenges associated with small sample sizes. Although we employed LOOCV and permutation testing to mitigate overfitting, the risk of spurious associations remains elevated in such low-sample regimes. Future validation in larger, multi-institutional cohorts is essential before clinical translation.

Furthermore, our binary classification approach (one-year survival vs. death) does not leverage time-to-event information. While survival-aware models like Cox regression or deepSurv could offer richer insights, they are generally unsuitable for cohorts of this size due to high parameter demands and instability. This represents a methodological trade-off between clinical simplicity and temporal granularity.

In this study, we primarily compared our models with commonly used machine learning approaches because the available clinical information did not fully align with the variables typically included in established GBM prognostic tools such as clinical nomograms. As a result, a direct benchmark comparison could not be performed within the scope of the present dataset. Future work incorporating more comprehensive clinical and molecular parameters will enable such comparisons and further clarify the clinical relevance of our findings.

## 5. Conclusions

In this study, we investigated machine learning models using radiomics features from FMISO-PET and multiparametric MRI, along with clinical variables, for one-year survival prediction in glioblastoma (GBM) patients. Our results showed that the FMISO-based radiomic model achieved the best performance among all imaging modalities (AUC = 0.870), yet the clinical data-only model—comprising age, sex, and average FMISO SUV—yielded the highest overall predictive accuracy (AUC = 0.921). This suggests that, in small cohorts, readily available clinical factors may provide more robust prognostic signals than high-dimensional radiomic features, though the observed strong influence of sex warrants caution due to gender imbalance in our sample (23 males vs. 12 females).

Looking ahead, the clinical model could be integrated into routine postoperative workflows: within 48 h of surgery, a patient’s age, sex, and average FMISO SUV—derived from a standard hypoxia PET scan—could be input into the trained linear regression model via a secure interface to generate a personalized probability of one-year survival. This output may aid clinicians and patients in shared decision making regarding adjuvant therapy intensity, eligibility for clinical trials, or early palliative care planning. Nevertheless, prospective validation in larger, multicenter cohorts is essential to confirm the generalizability and clinical utility of these findings.

## Figures and Tables

**Table 1:** Patient demographics.

	Alive	Dead
Male	9	14
Female	10	2
Age (mean ± standard deviation)	53.4 ± 8.37	61.2 ± 6.35

**Table 2:** Summary of predictive performance of each modality.

Modality	Machine Learning Model	AUC [95% CI]	Accuracy	Precision	Recall	Permutation *p*-Value *
FMISO	SVM	0.645 [0.441, 0.853]	0.714	0.737	0.737	0.058
	LR	0.641 [0.444, 0.840]	0.743	0.778	0.737	0.080
	RF	0.870 [0.734, 0.970]	0.829	0.783	0.947	0.001
T1	SVM	0.796 [0.608, 0.945]	0.857	0.938	0.790	0.001
	LR	0.770 [0.576, 0.920]	0.829	0.842	0.842	0.007
	RF	0.743 [0.553, 0.902]	0.800	0.800	0.842	0.003
T2	SVM	0.862 [0.709, 0.985]	0.886	0.895	0.895	0.001
	LR	0.783 [0.602, 0.935]	0.857	0.850	0.895	0.002
	RF	0.803 [0.610, 0.953]	0.829	0.810	0.895	0.001
FLAIR	SVM	0.691 [0.477, 0.874]	0.800	0.773	0.895	0.023
	LR	0.684 [0.484, 0.863]	0.743	0.750	0.790	0.033
	RF	0.798 [0.618, 0.959]	0.771	0.824	0.737	0.002
Clinical	SVM	0.878 [0.742, 0.971]	0.829	0.842	0.842	0.001
	LR	0.921 [0.820, 0.986]	0.800	0.833	0.790	0.001
	RF	0.842 [0.673, 0.976]	0.857	0.850	0.895	0.001
Combined all SFS model	SVM	0.834 [0.661, 0.966]	0.743	0.812	0.684	0.001
	LR	0.793 [0.595, 0.947]	0.857	0.850	0.895	0.003
	RF	0.862 [0.701, 0.989]	0.886	0.895	0.895	0.001

AUC: Area Under Curve, CI: Confident Interval, LR: Linear Regression, RF: Random Forest, SFS: Sequential Forward Selection, SVM: Support Vector Machine. * Permutation *p*-values < 0.01 were considered statistically significant.

## Data Availability

Datasets were acquired in the American College of Radiology Imaging Network 6684 (ACRIN 6684) imaging trial from The Cancer Imaging Archive (TCIA).

## Abbreviations

The following abbreviations were used in this article:

ACRIN 6684	American College of Radiology Imaging Network 6684
AUC	Area Under Curve
CI	Confidence Interval
FLAIR	Fluid-Attenuated Inversion Recovery
FMISO	Fluoromisonidazole
GBM	Glioblastoma Multiforme
GLCM	Gray-Level Cooccurrence Matrix
GLDM	Gray-Level Dependence Matrix
GLNU	Gray-Level Non-Uniformity
GLRLM	Gray-Level Run Length Matrix
GLSZM	Gray-Level Size Zone Matrix
HIF	Hypoxia-Inducible Factor
KPS	Karnofsky Performance Status
LOOCV	Leave-One-Out Cross-Validation
LR	Linear Regression
M2DS	Maximum 2D Diameter Slice
MDR1	Multidrug Resistance 1
ML	Machine Learning
MR	Magnetic Resonance
MRI	Magnetic Resonance Imaging
NGTDM	Neighborhood Gray Tone Difference Matrix
PET	Positron Emission Tomography
RE	Run Entropy
RF	Random Forest
ROC	Receiver-Operating Characteristics
ROI	Region Of Interest
RT	Radiation Therapy
SFS	Sequential Forward Selection
SUV	Standardized Uptake Value
SVM	Support Vector Machine
SZNU	Size Zone Non-Uniformity
TCIA	The Cancer Imaging Archive
TMZ	Temozolomide
